# Reoptimization of single-joint motor patterns to non-Earth gravity torques induced by a robotic exoskeleton

**DOI:** 10.1016/j.isci.2023.108350

**Published:** 2023-10-28

**Authors:** Dorian Verdel, Simon Bastide, Franck Geffard, Olivier Bruneau, Nicolas Vignais, Bastien Berret

**Affiliations:** 1 Université Paris-Saclay, CIAMS, 91405 Orsay, France; 2 CIAMS, Université d’Orléans, Orléans, France; 3 CEA List, 91120 Palaiseau, France; 4 LURPA, Mechanical Engineering Department, ENS Paris-Saclay, Université Paris-Saclay, 91190 Gif-sur-Yvette, France; 5 Institut Universitaire de France, Paris, France

**Keywords:** Aerospace Engineering, Control engineering

## Abstract

Gravity is a ubiquitous component of our environment that we have learned to optimally integrate in movement control. Yet, altered gravity conditions arise in numerous applications from space exploration to rehabilitation, thereby pressing the sensorimotor system to adapt. Here, we used a robotic exoskeleton to reproduce the elbow joint-level effects of arbitrary gravity fields ranging from 1g to −1g, passing through Mars- and Moon-like gravities, and tested whether humans can reoptimize their motor patterns accordingly. By comparing the motor patterns of actual arm movements with those predicted by an optimal control model, we show that our participants **(**N=61) adapted optimally to each gravity-like torque. These findings suggest that the joint-level effects of a large range of gravities can be efficiently apprehended by humans, thus opening new perspectives in arm weight support training in manipulation tasks, whether it be for patients or astronauts.

## Highlights

• Humans adapt to a wide range of gravity-like torques induced by a robotic exoskeleton
• Single-joint movements adapt during the first 15 trials even when gravity is inverted
• The kinematic and EMG patterns are consistently modulated across gravity conditions
• The adapted motor patterns are predicted by a smooth-effort optimal control model

## Graphical abstract

**Figure:**
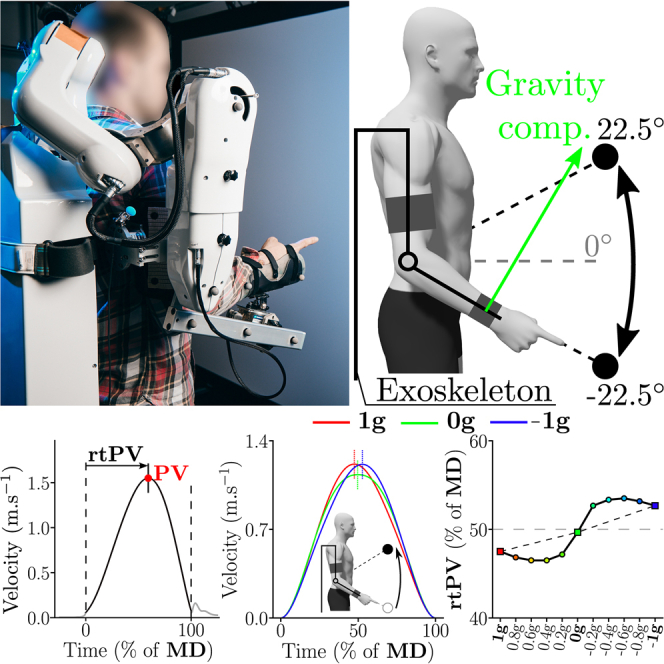

## Introduction

Earth’s gravity has pervasive effects on human neuromechanics and motor control. Several studies have suggested that our central nervous system (CNS) has an internal representation of gravity, spread over different brain areas, which allows to optimize the control of movement with respect to the ambient gravity field.^1^^,^^2^^,^^3^^,^^4^^,^^5^ The most direct evidence of such a gravity-exploitation theory came from studies conducted with astronauts in—or returning from—missions and during parabolic flights.^6^^,^^7^^,^^8^^,^^9^^,^^10^^,^^11^^,^^12^^,^^13^^,^^14^ Indirect evidence was also obtained by comparing the characteristics of vertical and horizontal movements.^10^^,^^11^^,^^15^^,^^16^^,^^17^^,^^18^^,^^19^^,^^20^^,^^21^ In particular, several studies reported consistent kinematic differences between vertical and horizontal movements, which gradually vanished through the adaptation to microgravity or could be recreated when applying a gravity-like force field in microgravity.^7^^,^^22^ This evidence was further supported by congruent observations of muscle patterns, which were found to substantially differ depending on motion direction with respect to gravity, in both humans and monkeys.^21^^,^^23^^,^^24^^,^^25^ Importantly, the adaptation of kinematic and muscular patterns to the ambient gravity field was found to comply with the predictions of optimal control models based on effort minimization. Several model-based studies supported the hypothesis that the CNS optimally exploits gravity during arm motor planning.^6^^,^^10^^,^^21^^,^^26^^,^^27^^,^^28^^,^^29^

Interestingly, the gravity-exploitation theory makes specific predictions in arbitrary gravity fields which have been untested so far. Studies in parabolic flights have allowed to test the theory in a couple of hypo- and hyper-gravity fields with a limited number of trials and participants.^6^^,^^10^^,^^13^^,^^14^ Moreover, while very relevant to space exploration,^30^^,^^31^^,^^32^^,^^33^^,^^34^ completely immersing participants in a novel gravity field is not representative of other applications. In rehabilitation, it is common to use devices to support a patient’s limb and reduce their muscle effort required to counteract gravity.^35^^,^^36^^,^^37^^,^^38^^,^^39^^,^^40^^,^^41^^,^^42^^,^^43^ In this case, the neuromechanical system is locally impacted, mostly through somatosensory information. In principle, this information could be sufficient to update the internal representation of gravity torques. Moreover, the somatosensory system has been shown to play a predominant role for learning new dynamics efficiently.^44^^,^^45^ Participants could thus adapt their motor planning to non-Earth gravity torques as predicted by the gravity-exploitation theory. Alternatively, participants could adapt to compensate the non-Earth gravity torques and preserve their nominal movement kinematics. In this case, either incongruent sensory signals could have prevented the reoptimization of movement according to the gravity-like torques or the gravity-exploitation theory must be revised to account for motor patterns in previously untested gravities.

**Figure 1:**
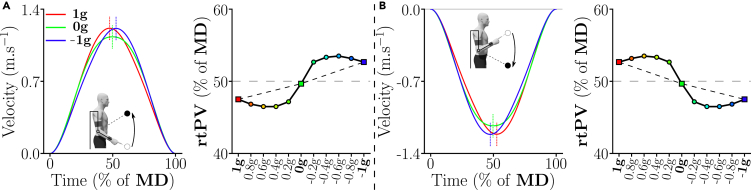
Predictions of the gravity-exploitation theory according to the Smooth-Effort (*SE*) optimal control model of Using a preexisting version of the SE model,^21^ we simulated fast pointing movements with the forearm of duration *MD* = 0.6s, while only varying gravity. In practice, in the present paper, the dynamics were slightly different due to the inertial behavior of the exoskeleton, which induced different rtPV values but a similar evolution trend (the reader is deferred to the STAR Methods for details). (A) Predicted upward velocity profiles for the 1g, 0g and −1g conditions. Vertical dashed lines indicate where the peak velocity (*PV*) is reached, which highlights the variations of the relative time-to-peak-velocity parameter (rtPV) according to the model. The right side of the panel exhibits the predicted evolution of rtPV for upward movements (in percentage of movement duration, *MD*) for 11 gravity torques ranging between 1g and −1g. While a linear gradient can be seen between the 1g, 0g, and −1g conditions, the finer-grained analysis reveals a non-linear evolution of rtPV as a function of gravity acceleration. (B) Predictions of velocity profiles and rtPV evolution for the same gradient of gravity torques for downward movements.

To test whether participants can reoptimize their motor patterns to arbitrary gravity-like torques at the joint level, the present paper leverages recent advances regarding arm weight compensation with active exoskeletons^46^ to induce gravito-inertial dynamics that were hitherto hardly achievable. Such modified gravito-inertial dynamics do not broadly impact all sensory systems (in particular the vestibular one) as would a complete immersion in a non-Earth gravity field. Nevertheless, they can give precious insights regarding the adaptation of human movement to novel joint dynamics mimicking non-Earth gravity fields. This follows a general path toward the exploitation of robotics in neuroscience.^47^ Here, we used the ABLE robotic exoskeleton^48^ to induce various gravity-like torques at the participant’s elbow, ranging from normal gravity (1g) to reversed gravity (−1g) and passing through microgravity (0g). A gradual change of gravity with a 0.2g-step ranging from −1g to 1g was also applied on the forearm of participants, thereby including Mars- and Moon-like gravity torques. The gravity-exploitation theory was then tested against experimental data for single-joint pointing movements. The predictions of a representative model, termed Smooth-Effort (*SE*) model,^21^ are illustrated in Figure 1. The changes in velocity profiles are quantified in terms of the relative time to peak velocity (rtPV), which is known to be a robust gravity-dependent parameter. This gravity-dependent asymmetry of velocity profiles for single-joint movements is well documented and is predicted to vary in the 1g, 0g, and −1g conditions if the gravity-like torque is exploited as a motive force. Furthermore, a striking non-linear evolution of rtPV is even predicted when varying gravity more finely because the exploitation of gravity depends on its actual effect on the limb’s acceleration for a fixed movement duration. If these theoretical predictions match experimental data, this would support the gravity-exploitation theory and indicate that local somatosensory information can be used to reoptimize human motor patterns as a function of the gravity-like torques induced by an exoskeleton.

## Results

**Figure 2:**
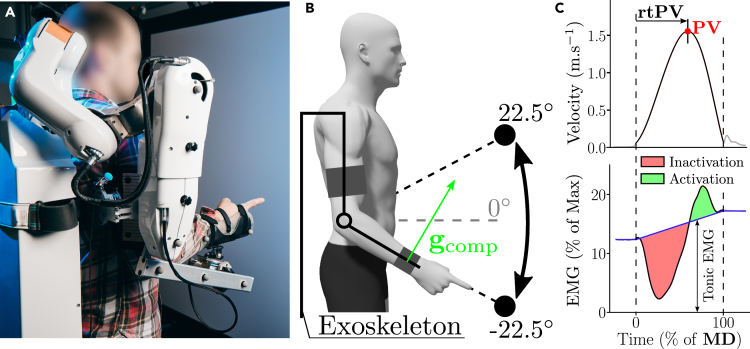
Experimental setup and main studied parameters (A) Illustration of a participant connected to the exoskeleton during the pointing task. (B) Schematic illustration of the motor task, consisting in forearm movements (here of $45^{\circ }$) with the human connected to the ABLE robotic exoskeleton. A force/torque sensor placed at the interface between the participant and the exoskeleton (about the wrist joint) allowed to track a desired normal force $g_{comp}$, mechanically corresponding to the effect of gravity-like torques ranging from 1g to −1g. The real-time forearm inclination and joint misalignments between the human and the robot were taken into account to accurately estimate $g_{comp}$.^46^ (C) Definition of the main kinematic and EMG parameters analyzed in the present study for an example upward movement in the −1g condition. Fast pointing movements are typically characterized by velocity profiles that mainly consist of one acceleration phase and one deceleration phase. Here, the temporal structure of movement was characterized by the rtPV parameter, as it is known to be sensitive to gravity.^10^ The represented EMG data are the envelope of the filtered, rectified and normalized signal recorded during −1g motions. Regarding EMG data, we systematically subtracted the tonic activity (blue line) from rectified EMGs and analyzed the resulting phasic EMG patterns. This phasic activity is also known to be sensitive to gravity.^21^ Negative phasic activity is referred to as inactivation and indicates periods where the EMG activity of a muscle is below the tonic level that would be required to counteract gravity (in shaded red). Positive phasic activity, referred to as activation (in shaded green), is responsible of net accelerations in the gravity field.

We asked N=61 participants to perform fast pointing movements of $45^{\circ }$ with the forearm toward semi-spherical targets of radius 2cm. The participants were connected to a robotic exoskeleton that was controlled to mechanically generate various gravity-like torques at the participant’s elbow joint. The control law was individualized thanks to a thorough identification procedure conducted prior to each experiment and validated in a previous study.^46^ The task is illustrated in Figure 2A and the reader is deferred to the STAR Methods for more details about the experimental procedures.

A series of three experiments was conducted in this work. In the first two experiments, three gravity-like torques (1g, 0g, −1g) were tested to analyze if and how participants changed their motor patterns according to the induced gravity-like torques (Experiment 1 and Experiment 2). In the last experiment, a gradient of 11 gravity-like torques, ranging from −1g to 1g with a step of 0.2g was tested to assess whether gradual changes of simulated gravity torques lead to nonlinear changes of motor patterns in agreement with the gravity-exploitation theory. The main kinematic and muscular parameters used to quantify motor patterns are defined in Figure 2B.

### Motor patterns change significantly with respect to gravity-like torques

In Experiment 1, participants (N=22) performed 6 consecutive blocks of 15 upward and 15 downward 45-degrees elbow movements in each of the 1g, 0g, −1g conditions. The order of the conditions was randomized. No evidence of adaptation was found across the 6 blocks performed in each condition of Experiment 1, for all the tested kinematic and EMG parameters (see Figure S1). Furthermore, no adaptation across blocks was found on parameters usually impacted by gravity (see Figure S2). As a consequence, all the subsequent analyses were conducted on data averaged across all the blocks, and we focus hereafter on the changes induced by the simulation of different gravity-like torques on the average motor patterns. The analyses were conducted separately for upward and downward movements, as they are known to be impacted differently by gravity efforts.^10^^,^^21^

#### Kinematic analysis

##### Upward movements

**Figure 3:**
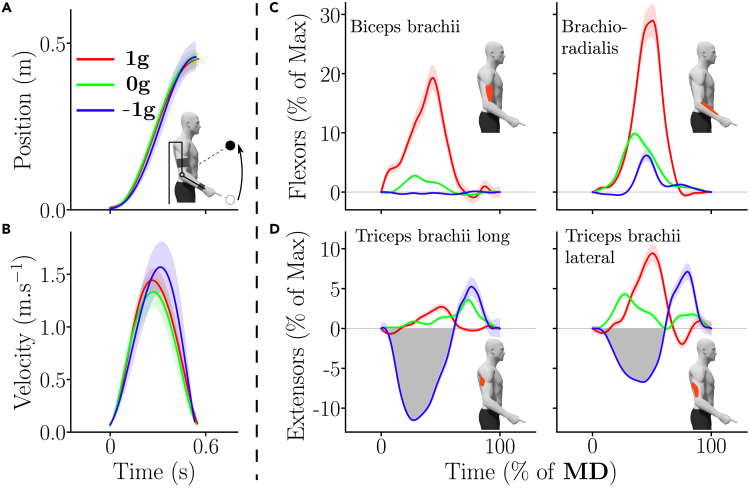
Kinematics and phasic EMG envelopes of upward movements for one representative participant in the different gravity conditions (red = 1g, green = 0g and blue = −1g) (A) Mean hand displacement. Standard errors across trials are represented as shaded areas. (B) Mean hand velocity profile. (C and D) Mean phasic flexors and extensors muscle patterns in the different gravity conditions, normalized in time (*MD* was about 0.6s). Notably, an early inactivation of the extensors (negative phasic area, emphasized by the gray shaded area) in the −1g condition can be observed (see blue trace in D).

Figure 3 depicts the average upward motor patterns (position and velocity, and phasic EMGs) for a representative participant in the 3 gravity conditions of Experiment 1.

As already mentioned, an interesting parameter, theoretically revealing gravity exploitation, and sensitive to the ambient gravity acceleration is rtPV.^10^ This parameter allows to compare kinematic motor patterns of movements of different durations. Here, only slight variations of durations and speeds were found between gravity conditions. Movements performed in −1g tended to be slightly faster than those performed in 0g and 1g, as reflected by a repeated-measures ANOVA conducted on *MD* (p=0.004, $F_{2,42}=6.72$, and $\eta^{2}=0.24$), but no difference on *MD* was found across conditions with post-hoc pairwise comparisons (p>0.18 for all comparisons). To further ensure that these time variations cannot explain the evolution trends of the rtPV predicted by the *SE* model, we performed simulations of movements in 1g for a large range of *MD* (see Figure S4). These simulations clearly show that these time variations cannot predict the same sigmoidal evolution of the rtPV as does the modulation of gravity acceleration.

**Figure 4:**
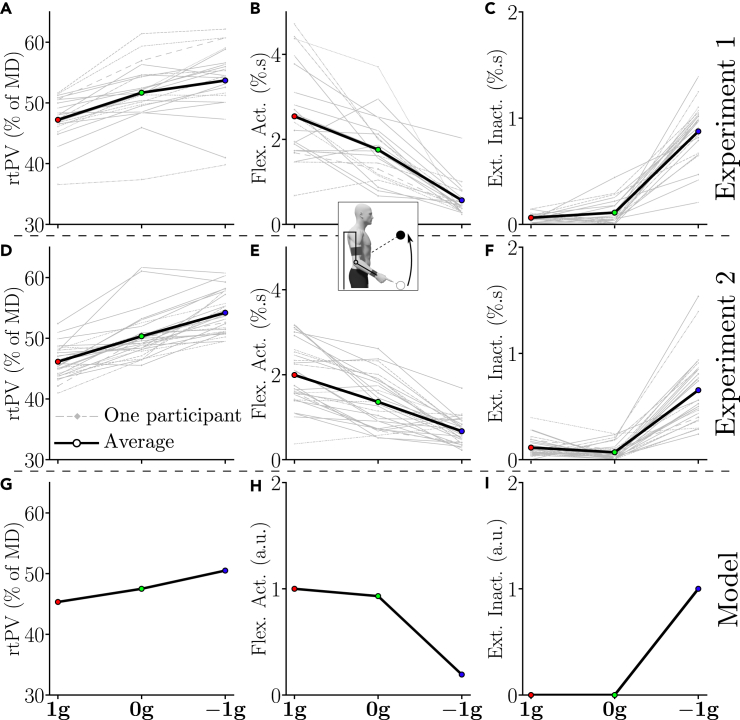
Average kinematic and muscular behavior of each participant (gray) and of the tested population (black bold) for upward movements of both Experiment 1 and Experiment 2 (A, and D) Adaptation of rtPV across conditions. (B and E) Adaptation of flexors burst area across conditions.(C and F) Adaptation of extensors inactivation area across conditions. (G) Predicted rtPV. (H) Predicted positive phasic torque area (normalized by maximum). (I) Predicted negative phasic torque area (normalized by maximum).

Regarding rtPV, group and individual data are depicted in Figure 4A, revealing a main effect of the gravity-like torque on rtPV during upward movements (repeated-measures ANOVA: p<0.001, $F_{2,42}=52.6$ and $\eta^{2}=0.71$). Post-hoc analyses indicated that the rtPV of upward movements was significantly higher in the 0g and −1g conditions when compared with the 1g condition (p<0.002 in both cases). This means that upward velocity profiles were more left-skewed in 0g and −1g than in 1g. However, no difference was found between the 0g and the −1g conditions in this sample (p=0.225).

To self-replicate our findings and test whether significance can be reached for comparisons between 0g and −1g, we performed an additional experiment including two blocks of 25 upward movements of $45^{\circ }$ for each gravity condition, knowing that adaptation was very quick (referred to as Experiment 2, N=29 participants). The rtPV values obtained in this experiment are depicted in Figure 4D.

The same trends as in Experiment 1 were observed. Repeated-measures ANOVA confirmed a main effect of local gravity on rtPV during upward movements (p<0.001, $F_{2,56}=93.9$, and $\eta^{2}=0.77$). Post-hoc tests further revealed significant differences between all gravity conditions in this sample (p<0.001 in all cases).

##### Downward movements

**Figure 5:**
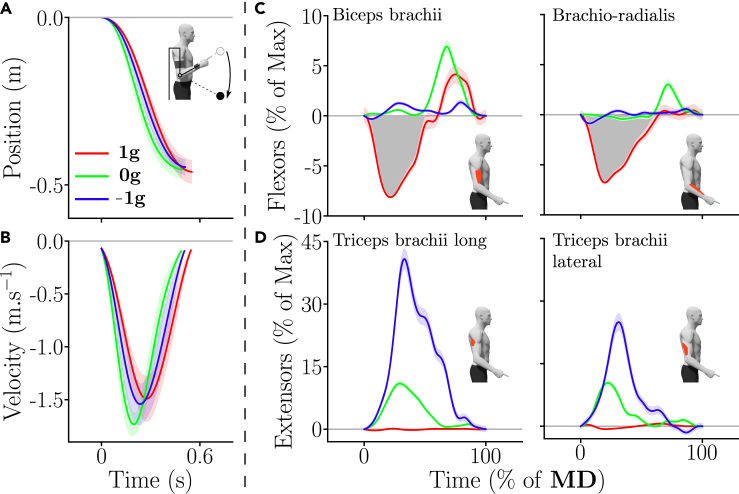
Kinematics and phasic EMG envelopes of downward movements for one representative participant in the different gravity conditions (red = 1g, green = 0g and blue = −1g) (A) Mean hand displacement. Standard errors across trials are represented as shaded areas. (B) Mean hand velocity profile. (C and D) Mean phasic flexors and extensors muscle patterns in the different gravity conditions, normalized in time (*MD* was about 0.6s). Notably, an early inactivation of the flexors (negative phasic area, emphasized by the gray shaded area) in the 1g condition can be observed (see red trace in C).

Figure 5 depicts the average downward motor patterns (position and velocity, and phasic EMGs) for a representative participant in the 3 gravity conditions of Experiment 1.

**Figure 6:**
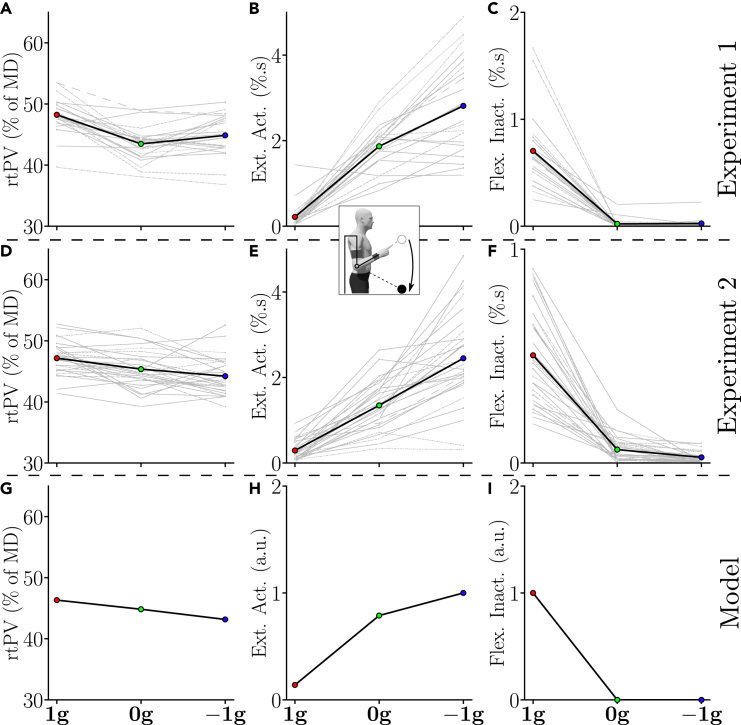
Average kinematic and muscular behavior of each participant (gray) and of the tested population (black bold) for downward movements of both Experiment 1 and Experiment 2 (A and D) Adaptation of rtPV across conditions. (B and E) Adaptation of extensors burst area across conditions. (C and F) Adaptation of flexors inactivation area across conditions. (G) Predicted rtPV. (H) Predicted positive phasic torque area (normalized by maximum). (I) Predicted negative phasic torque area (normalized by maximum).

The same important kinematic parameter as for upward movements (i.e., rtPV), was also analyzed for downward movements. The group and individual data obtained on this parameter are depicted by Figures 6A and 6D for Experiment 1 and Experiment 2, respectively. A main effect of the condition on rtPV during Experiment 1 was revealed using a repeated-measures ANOVA (p<0.001, $F_{2,42}=30.2$ and $\eta^{2}=0.59$). Post-hoc analyses revealed that the rtPV of downward movements was significantly lower in the 0g and −1g conditions than in the 1g condition (p<0.002 in both cases). Furthermore, a main effect of the condition on rtPV was also observed during Experiment 2 (repeated-measures ANOVA: p<0.001, $F_{2,56}=15$ and $\eta^{2}=0.35$). Again, post-hoc analyses exhibited a significantly lower rtPV in the 0g and −1g conditions than in the 1g condition (p<0.02 in both cases). However, no significant difference was found between the 0g and −1g conditions in terms of rtPV (p>0.12 in both cases).

Together, the results of Experiment 1 and Experiment 2 on both upward and downward movements clearly show that the averaged velocity profiles were robustly tuned according to the gravity-like torque created by the exoskeleton. Furthermore, this tuning was consistent with the predictions of the *SE* model (see Figures 4G and 6G). Finally, the absence of significant difference between the 0g and −1g conditions in terms of rtPV for downward movements was consistent with the model predictions. Indeed, the model predicted smaller inter-condition differences for these movements due to the specific dynamics of the exoskeleton (see STAR Methods for details).

To better understand the cause of this gravity-dependent tuning of velocity profiles, we then investigated the underlying muscular strategies. The patterns of activation of flexor and extensor muscles may reveal the extent to which participants took advantage of the novel gravity-like torque.^21^^,^^23^^,^^24^^,^^25^

#### Muscular analysis

Forearm movements are controlled by opposing muscle groups that can be gathered as follows: (1) flexors (e.g., mainly the biceps-brachii and brachio-radialis) and (2) extensors (e.g., mainly the triceps-brachii long and lateral heads). In the Earth’s gravity field, the flexors are antigravity muscles in the sense that they generally allow counteracting the action of gravity. The extensors play the opposite role in 1g and could be termed gravity muscles. In 0g this distinction is irrelevant whereas in −1g the role played by flexors and extensors should be inverted such that extensors should become antigravity muscles. Consistently with the kinematic analyses, the muscle patterns were first assessed for upward movements and then for downward movements.

##### Upward movements

Qualitatively, the upward EMG patterns are depicted in Figure 3C and 3D for each gravity condition for a representative participant. In the 1g condition, the movement was triggered by a strong phasic activation of the flexors, which was associated with some co-activation of the extensors as commonly observed.^21^ In 0g, the EMG patterns were similarly structured as in 1g, except that the activation of the flexors was weaker. Finally, the EMG pattern in the −1g condition was very different and started with an inactivation of the extensors, meaning that the participants let the gravity-like torque created by the robot initiate the upward movement. This inactivation was followed by an activation of the extensors for decelerating the movement at the end. This negativity existed and was adapted during the first block (see Figure S2C).

These qualitative observations were supported by quantitative analyses performed on the activation of flexors and the inactivation of extensors in the different conditions (see Figures 4B, 4C, 4E, and 4F). As for the kinematic analysis, both group and individual data are reported. There were clear differences between the different gravity conditions in terms of flexors activation (see Figures 4B and 4E; main effect, p<0.0041, $F_{2,42}=45.6$ and $\eta^{2}=0.68$). Post-hoc analyses indicated that all gravity conditions were different (p<0.001 in all cases). In particular, there was a 30% decrease in flexors activation between the 1g and 0g conditions and a 68% decrease in flexors activation between the 0g and −1g conditions during Experiment 1. The same significant decrease of flexors activation was observed during Experiment 2 between the three tested conditions (main effect: p<0.0041, $F_{2,56}=59.9$ and $\eta^{2}=0.68$; post-hoc: p<0.003 in all cases).

The inactivation of extensors also exhibited clear differences between the conditions (see Figures 4C and 4F; main effect: p<0.001, $F_{2,42}=147$ and $\eta^{2}=0.87$). In particular, during Experiment 1, upward movements in the −1g condition were the only ones that exhibited clear extensors inactivation, which was reflected in post-hoc comparisons with the 1g and 0g conditions (p<0.001 in both cases). The same trend was observed during Experiment 2 (main effect: p<0.001, $F_{2,56}=83.1$ and $\eta^{2}=0.75$). The upward movements performed in the −1g condition was again the only ones exhibiting extensors inactivation (post-hoc: p<0.001 in both cases).

##### Downward movements

Qualitatively, the downward EMG patterns are depicted in Figures 5C and 5D for each gravity condition for a representative participant. These patterns mirrored those obtained for upward movements. In the 1g condition, downward movements were initialized by a strong inactivation of the flexors and were stopped through a braking activation of these same muscles, as commonly observed.^21^ Interestingly, in the 0g and −1g conditions, the flexors inactivation vanished and the movement was initialized by an activation of the extensors, which tended to be stronger in the −1g condition. Furthermore, in the 0g condition, the movement was stopped by a clear braking activation of the flexors. On the contrary, in the −1g condition, the participant seemed to take advantage of the upward gravity-like torque to brake the movement, which induced a very low activity of the flexors.

These qualitative observations were supported by quantitative analyses performed on the activation of extensors and the inactivation of flexors in the different conditions (see Figures 6B, 6C, 6E, and 6F). As for the kinematic analysis, both group and individual data are reported. There were clear differences between the different gravity conditions in terms of extensors activation (see Figures 6B and 6E; main effect, p<0.001, $F_{2,42}=87.7$ and $\eta^{2}=0.81$). Post-hoc analyses indicated that all conditions were different (p<0.001 in all cases). In particular, there was a 780% increase in extensors activation between the 1g and 0g conditions and a 50% increase in extensors activation between the 0g and −1g conditions during Experiment 1. The same significant increase of extensors activation was observed during Experiment 2 between the three tested conditions (main effect: p<0.001, $F_{2,56}=96.3$ and $\eta^{2}=0.77$; post-hoc: p<0.001 in all cases).

The inactivation of flexors also exhibited clear differences between the conditions (see Figure 6C and 6F; main effect: p<0.001, $F_{2,42}=83.4$ and $\eta^{2}=0.8$). In particular, during Experiment 1, the 1g condition was the only one that exhibited clear flexors inactivation, which was reflected in post-hoc comparisons with the 0g and −1g conditions (p<0.001 in both cases). The same trend was observed during Experiment 2 (main effect: p<0.001, $F_{2,56}=119$ and $\eta^{2}=0.81$). The 1g condition was again the only one exhibiting flexors inactivation (post-hoc: p<0.001 in both cases).

In summary, these two first experiments revealed that the participants reoptimized their motor patterns toward effort minimization according to the gravity-like torques induced by the exoskeleton, in agreement with the prediction of the gravity-exploitation theory. Indeed, as previously stated, the skewness of velocity profiles changed significantly in the 0g and −1g conditions compared to the 1g condition as predicted by the *SE* model, and coherent changes in muscle activation and inactivation patterns were observed. Furthermore, the normalized phasic torque areas predicted by the *SE* model exhibit a similar evolution with regard to the applied gravity-like torque, which strengthens the gravity-exploitation hypotheses (although they are only correlated and not quantitatively comparable). In particular, for upward movements, a reduction of the positive torque area (which can be correlated with flexors activation) was predicted, although the torque reduction was smaller in 0g than in the EMG data. The residual activation of flexors in −1g, allowing to accelerate more than by just letting the robot torque perform the acceleration, was also predicted. Finally, the strong inactivation of extensors in −1g, allowing to take advantage of the gravity-like torque to accelerate the movement, was also predicted by the model as a negative phasic torque of the extensors. In the case of downward movements, the increase in extensors activation between 1g, 0g, and −1g was predicted by the model. Furthermore, the strong inactivation of the flexors to initialize the movement in the 1g condition was also predicted.

To further test the striking prediction of a non-linear evolution of rtPV when gravity is varying more finely, we conducted a third experiment where we gradually varied the gravity compensation with a 0.2g-step.

### Adaptation to gradual changes of gravity-like torques conforms to the gravity-exploitation theory

In Experiment 3 (N=10 participants) we considered the same 45-degrees elbow flexions and extensions for a gradient of 11 gravity-like torques, equally spaced between 1g and −1g, and passing through 0g, Mars-like gravity (about $3.7m\text{.}s^{-2}$, which is around 0.4g) and Moon-like gravity (about $1.6m\text{.}s^{-2}$, which is slightly under 0.2g).

**Figure 7:**
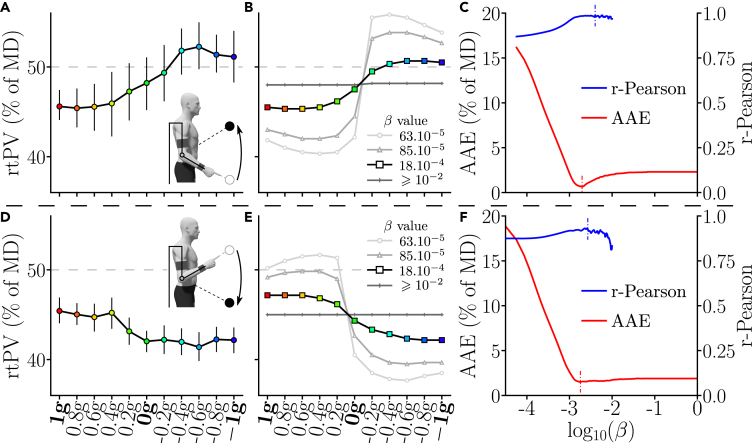
Averaged rtPV adaptation and simulations (A and D) Experimental results of Experiment 3 for upward movements (A) and downward movements (D). Averaged data are represented with bars representing the standard error. (B and E) Upward (B) and downward (E) simulation results for various weighting of the cost function. The simulation result highlighted corresponds to the optimal weighting of the cost function in terms of average absolute error (AAE). (C and F). Upward (C) and downward (F) evolution of the AAE and of the Pearson correlation coefficient with regard to the cost function weighting with $\beta \in \left[10^{-5},1\right]$. The Pearson correlation coefficient was only computed when the predicted rtPV presented enough variability across conditions (not defined otherwise) and plotted for significant correlations (p<0.05). The optimal values of β with regard to each of these error criteria are highlighted with small dotted bars. B, C, E, and F. These graphs allow to exhibit the evolution of the prediction quality both in terms of gradient and average value with regard to the simulated compromise between smoothness and effort.

Results for rtPV are depicted in Figures 7A and 7D. Data for upward movements showed that rtPV globally tended to increase from 1g to −1g, hence confirming our previous observations. This gradual increase exhibited a non-linear trend on average. The average rtPV varied according to a “sigmoidal” form across the different gravity conditions, with a minimum observed in 0.8g and a maximum observed in −0.6g. Furthermore, between 1g and 0.2g the average rtPV followed a smooth gradient but stayed clearly under 50% of *MD*, which implies that the acceleration phase was shorter than the deceleration phase (right-skewed velocity profiles). The average rtPV observed in 0g and −0.2g were close to 50% of *MD*, the acceleration and deceleration phases were therefore almost equivalent (approximately symmetric velocity profiles). Finally, between −0.4g and -1g, the rtPV was above 50% of *MD*, which implies that the acceleration phase was on average longer than the deceleration phase (left-skewed velocity profiles). Data for downward movements also showed a sigmoidal evolution through the different gravity conditions. This sigmoidal form was mirroring the observations on upward movements, which means that rtPV decreased when the gravity-like torque varied from 1g to −1g.

These trends were reminiscent of the ones predicted by the *SE* model given in Introduction (see Figure 1), representative of the gravity-exploitation theory.^10^^,^^21^ This model allows to simulate movements under the assumption of the minimization of a compromise between the absolute work of muscle torques and the time-integral of a smoothness term, which corresponds to an effort minimization model with smoothness regularization. In its present form, the model had one free parameter in the cost function, which sets the compromise between the effort and smoothness terms (β in Equation 5). Predictions of the *SE* model in terms of rtPV have already been exemplified in Figure 1A, which can be compared qualitatively to Figure 7B. However, the predictions exhibited in Figures 7B and 7E are centered differently from those of Figure 1, which is due to the specific inertial dynamics induced by the exoskeleton (see STAR Methods and Figure S3 for details). Here, the quality of the model prediction was assessed more quantitatively by computing the average absolute error (AAE) and the Pearson correlation coefficient between the experimental and simulated rtPV. The goal was to evaluate the extent to which the gravity-dependent tuning of this parameter was captured by the model.

Furthermore, by varying β, we could thus seek for an optimal weighting of the cost function to match the experimental data, which corresponds to an inverse optimal control approach (e.g., identifying the weighted cost function that best fits experimental data). The results of this procedure are depicted in Figures 7B, 7C, 7E, and 7F. For upward movements, the *SE* model was able to provide both small estimation errors on rtPV (AAE ≈0.67% of *MD* with an optimal $\beta =19\times 10^{-4}$) and high Pearson correlation coefficients (*r*-Pearson >0.98 with an optimal $\beta =32\times 10^{-4}$). Similarly, for downward movements, the *SE* model provided both small estimation errors on rtPV (AAE ≈1.5% of *MD* with an optimal $\beta =17\times 10^{-4}$) and high Pearson correlation coefficients (*r*-Pearson >0.93 with an optimal $\beta =27\times 10^{-4}$). Furthermore, average unifying values of β for AAE and Pearson correlation would be $\beta =18\times 10^{-4}$ and $\beta =29\times 10^{-4}$, respectively. For both upward and downward movements, these unifying values resulted in small AAE errors (0.68% of *MD* and 1.5% of *MD*, respectively) and high Pearson correlation coefficients (0.985 and 0.915, respectively). Models with too small or too large β either predicted overestimated or underestimated changes of rtPV with respect to the gravity-like torque. Interestingly, the unifying values of β were close to those reported in previous works to predict the kinematics of 1° of freedom shoulder movements,^10^ which shows a certain consistency of the model predictions across different experimental paradigms.

## Discussion

In the present paper, we tested the gravity-exploitation theory by investigating if and how humans can reoptimize their motor patterns to arbitrary gravity-like torques locally induced by a robotic exoskeleton at the elbow joint. Kinematic and muscle patterns were thoroughly analyzed during pointing movements involving the forearm while the exoskeleton applied various gravity-like torques reproducing 0g (weightlessness), 0.4g (close to Mars’ gravity) or −1g (inverted Earth’s gravity), on the participant’s joint. In a series of three experiments, we found that participants changed their single-joint motor patterns according to the gravity-like torque, and tended to take advantage of it whenever possible. Indeed, varying the gravity-like torque from 1g to −1g was concomitant with a shift of hand velocity profiles from right-skewed to left-skewed during upward movements and vice versa during downward movements. Furthermore, EMG patterns were in line with an exploitation of the gravity-like torque, in both direction and magnitude, to accelerate the limb. This was particularly exemplified by the fact that upward movements systematically started with an inactivation of the extensors in −1g, which mirrored the inactivation of flexors triggering downward movements in 1g. These empirical observations were in good agreement with the predictions of an optimal control model based on effort minimization, thereby supporting the gravity-exploitation theory even when only reproducing its joint-level effects. In the following section, we discuss these findings with respect to the update of the internal model that could allow participants to reoptimize their motor patterns to novel gravity-like torques from somatosensory information.

Our participants changed their motor patterns in 0g and −1g. Block-wise analyses showed that plateaus on all the main kinematic parameters were reached after the first block performed in each condition, as shown in the Figures S1 and S2 and corresponding statistical analyses. A similar conclusion had been drawn with the same exoskeleton when increasing the apparent inertial torque without affecting the participants’ gravity torque.^49^ However, such an adaptation within a dozen trials may contrast with other works showing a slower adaptation to microgravity for similar single-joint arm movements, which took about 75 movements during parabolic flights to remove directional asymmetries in velocity profiles.^10^ The main difference between these studies seems to lie in the nature of the sensory changes induced by the novel environment. During parabolic or space flights, the sensory changes are global and captured by both the somatosensory and vestibular systems,^8^^,^^14^ which may imply a significant sensory re-weighting.^50^ Furthermore, it was shown that perceiving the vertical orientation was possible from proprioception within three weeks after bilateral vestibular loss^51^ or several parabolas in microgravity.^12^ Such a sensory re-weighting when vestibular information is altered may thus induce a longer update of the internal model of gravity. This process might conceivably be necessary to elicit a fine reoptimization of movement planning in a novel gravitational environment.^10^^,^^52^^,^^53^ During the present human-exoskeleton interaction, only somatosensory signals were modified through the contact with the exoskeleton. In this case, motor planning was adapted despite incongruent gravity-related sensory cues. Indeed, visual and vestibular cues were unchanged unlike muscle proprioception at the elbow joint when the robot applied a gravity-like torque. On the one hand, these results are consistent with the predominant role of the somatosensory system in the early learning of new dynamics on Earth.^44^^,^^45^^,^^54^ Here, the induced gravity-like torques essentially corresponded to a parametric change in the forearm’s dynamics, a type of alteration which is known to lead to relatively fast adaptations.^55^^,^^56^ In principle, this parametric change in the forearm dynamics could be estimated from somatosensory cues collected in statics before the movement.^57^ This feature could explain why the CNS can easily handle object manipulation on Earth,^58^ a familiar action in daily life which is similar to the present interaction task except that both inertia and gravity are changed when carrying real objects. On the other hand, studies on the adaptation of grip dynamics to altered gravito-inertial environments showed that somatosensory (haptic) feedback may not be sufficient to readily adjust object manipulation forces.^59^^,^^60^^,^^61^ Participants have been shown to struggle to adapt during a force reproduction task when exposed to non-Earth gravity fields during parabolic flights.^62^^,^^63^ Therefore, somatosensory information does not always allow an adaptation in one block as reported in this article, especially when the vestibular system is altered. This observation might be extended to vision. Indeed, when generalizing to interception tasks where the physical law of motion of external objects is also of concern, conflicting visual (0g) and somatosensory (1g) information can result in a consolidation of 1g movement timings, thereby limiting the adoption of correct 0g timings to appropriately fulfill the task.^64^ Actually, vision may strongly influence the planning of vertical movements similar to the present ones, possibly causing the adoption of non-optimal motor patterns.^15^^,^^65^ Furthermore, the considered task might have an impact on the adaptation because of different underlying neural processes.^66^

Here, the adaptation exhibited the signature of effort-based optimality with respect to the gravity-like torque induced by the exoskeleton. The asymmetry of velocity profiles (e.g., rtPV) was shown to change consistently across the different gravity conditions in all three experiments. During upward movements, the relative duration of the acceleration phase tended to increase when the simulated gravity decreased from 1g to −1g and this evolution was not simply linear. Conversely, during downward movements, the relative duration of the acceleration phase tended to decrease when the induced gravity increased, which mirrored the evolution observed during upward movements. All these changes were well predicted by the Smooth-Effort optimal control model (see Figures 7A–7C). For example, the maximum rtPV was reached around the −0.6g condition for most participants and then it started to decrease (see Figure 7A), in agreement with model predictions. Furthermore, when gravity was reversed and pushed the participant’s forearm upward, longer relative duration of acceleration were observed, thereby increasing the rtPV parameter. This particular kinematic strategy has been observed previously during downward motions in the Earth’s gravity field^6^^,^^10^^,^^11^^,^^17^^,^^18^^,^^19^^,^^20^^,^^21^ and was well explained by the gravity-exploitation theory.^10^^,^^21^^,^^26^ In particular, from the condition −0.4g, the average rtPV exceeds 50% of *MD* in Experiment 3. Interestingly, this strategy contrasts with accuracy constraints typically associated with rapid movements as it is known that deceleration is instead larger when participants are required to be maximally fast and accurate.^67^ Therefore, the left-skewness of velocity profiles was clearly not due to accuracy concerns but proved to be compatible with an optimal movement strategy taking into account the current gravity acceleration to minimize effort. Overall, the fact that results for downward movements mirrored those obtained for upward movements in the three experiments was very consistent with the predictions of the *SE* model and shows its robustness when varying movement direction. At the muscle level, changes in activation and inactivation patterns were also compatible with an exploitation of the gravity-like torque (e.g., 1g, 0g, and −1g conditions). The most notable effect is likely the adaptation of extensors to the −1g condition. In this condition, the movement was initiated with an inactivation of the extensors rather than with an activation of the flexors (see Figures 4C and 4D). This behavior is particularly revealing of the human capacity to optimally exploit the gravity-like torque. Indeed, even though an inverted gravity-like torque is very unusual, the participants were all able to take advantage of its presence to accelerate their limb upward with low effort (see Figures 4B and 4E). In the Earth’s gravity field, which in the present paper corresponds to the 1g condition, this inactivation pattern is usually observed on the flexors, which allows to use gravity to accelerate downward movements at their initiation.^21^^,^^24^^,^^25^ Here, this inactivation pattern to initialize downward movements was also observed. Consequently, we show that all the participants were able to assign to their extensors a role normally assigned to their flexors. This was probably possible thanks to the somatosensory information collected in statics before the movement.^57^ Overall, our experimental and computational analyses agree with the gravity-exploitation theory and not with an alternative gravity-compensation theory, which would predict that gravity-like torques should not affect kinematic motor patterns. The latter strategy could be implemented by means of a phasic activity controlling inertia-related efforts combined with a tonic activity systematically counteracting gravity-related efforts.^17^^,^^68^^,^^69^ In our study, it was clear that the participants did not attempt to counteract the force applied by the exoskeleton and maintain an unchanged kinematic strategy for all gravity-like torques. The observed changes of motor patterns were not arbitrary either, as they could have been if the system had failed to account for the new gravity-like torques during motor planning. Instead, our results point to a specific and optimal-like adaptation to each gravity-like torque induced by the exoskeleton.

It is worth stressing that different models could have been used as representatives of the gravity-exploitation theory. Here, we used a previously proposed model based on the minimization of the absolute work of muscle torques.^26^ We did not consider complex muscle dynamics to reduce the number of unknown parameters and simplify the modeling. Hence, only one parameter was left free in the model, which served to set the compromise between effort and smoothness optimization. Using an inverse optimal control approach,^27^ we identified the best-fitting β coefficient to replicate the data, which was relatively close to that proposed in previous studies. This analysis revealed that minimizing effort too strongly tended to overestimate the effects of gravity on the kinematics whereas maximizing smoothness tended to underestimate them. It could be interesting in the future to investigate the effects of using more accurate muscle models to refine the estimation of energy expenditure in such tasks.^70^ Nevertheless, conducting a sensitivity analysis related to the uncertainty about the various model parameters could be a tedious task for such optimal control simulations. Here, because we mainly wanted to examine how gravity can be exploited in motor planning, we examined a simpler model that cannot impute the gravity-dependent kinematic changes to the low-level contraction properties of muscles, in accordance with previous interpretations.^10^^,^^15^^,^^18^^,^^21^ Yet, studying longer term adaptations to altered gravity environment could provide an interesting avenue for future research because major neuromuscular reorganizations may then occur,^71^ which could strongly affect motor patterns. Alternatively, estimating metabolic energy through gas exchanges like in other studies^72^^,^^73^ could be interesting to estimate the different costs of movement in the various gravity environments and check whether a minimum is attained in the 0g condition for instance, or whether the costs decrease during longer-term adaptations. Finally, it is worth mentioning that our modeling did not attempt to account for movement duration and trial-by-trial variability. For the first point, the cost function could easily integrate a cost of time. It is indeed common to model a time-effort tradeoff in reaching movements to predict movement vigor.^74^^,^^75^^,^^76^ For the second point, sensorimotor noise could be included in the model to account for sensorimotor variability, in the spirit of the stochastic optimal control frameworks.^6^^,^^77^^,^^78^^,^^79^ The consideration of such features of movement planning and execution could be investigated in future works so as the extension to movements involving multiple degrees of freedom. In this case, active exoskeletons could be helpful to test the influence of various gravity-like torques on duration and variability as it allows to collect data more easily than during a complete immersion in a non-Earth gravity field.

Understanding how the human CNS integrates gravity in movement control has numerous applications. In particular, this question is critical to space exploration with astronauts.^53^^,^^80^^,^^81^^,^^82^^,^^83^ There, the use of active exoskeletons could be envisioned during long-term missions as a way to limit muscle atrophy^84^^,^^85^^,^^86^ (recreating Earth’s gravity torques in spaceships as a countermeasure) or as a way to train astronauts to perform dexterous manipulation tasks in specific gravity environments.^7^^,^^30^ Furthermore, weight compensation for patients suffering from muscle weakness is an appealing approach in neurorehabilitation. For example, post-stroke patients can recover more mobility when gravity-related efforts are compensated by a mechanical device.^35^^,^^36^^,^^37^^,^^38^^,^^39^ The present paper suggests that a clever use of gravity-like torques could be useful to facilitate arm movements in different directions, depending on their acceleration or deceleration phase. Playing with gravity-like torques could thus yield assistive control laws that are easily integrated by the human sensorimotor system. Therefore, locally varying gravity torques with a robotic exoskeleton may be an interesting approach for all applications where adapted weight support is relevant.

### Limitations of the study

Our current conclusions were obtained with a robotic exoskeleton controlled in such a way that a large range of gravity-like torques could be generated at the level of the human elbow joint. Although a subject-specific calibration was conducted to create the desired gravity-like torque as accurately as possible, there are currently two limits that should be mentioned. The first one is that exoskeletons may disturb the human motor behavior even in transparent mode (our 1g condition here), although interaction efforts are minimized. We have quantified the nature of the perturbation for elbow flexions/extensions in previous works and the remaining disturbances for upward movements were mainly due to the additional inertia caused by the exoskeleton.^46^^,^^87^^,^^88^^,^^89^^,^^90^ It could be possible to compensate this inertia by using predictive control methods.^91^^,^^92^ However, the inherent variability of human movement makes difficult the design of a simple and straightforward compensation method for human-exoskeleton interaction. One alternative, on which the present paper is based, is to assess the nature of the perturbation introduced by the exoskeleton *a posteriori* and integrate it in the models as an augmented inertia for the coupled human-robot system. The second limitation lies in the tracking of the desired gravity-like torque. Residual errors will inevitably occur due to inherent limits of the tracking control law. These errors were also quantified in a previous paper.^46^ Although relatively small, slight variations from a true gravity-like torque were present and it is difficult to estimate the extent to which it could affect the participants’ behavior. In particular, during upward movements, a homogeneous and slight increase in the apparent inertia is observable in Figure S3B. During downward movements, however, we found an increased resistance when compared to the gravity-like torque at the initiation of movement, which was explained by the specific dynamics of our exoskeleton. Once integrated in the model, experimental rtPV could be well predicted (see Figures 7D and 7E). Therefore, the imperfect behavior of the robot can likely yield motor patterns that differ from what would have been observed after steady adaptation to real Moon’s or Mars’ environments. However, substantial changes in the rtPV parameter were systematically observed across a large range of gravity-like torques, showing that the motor response to the perturbation depended clearly on the simulated gravity-like torque. Yet, whether the observed adaptation is due to the CNS adapting to an external perturbation interpreted as coming from a gravity-like field or just as a predictable/exploitable force field remains unclear. It would be interesting to compare if a similar adaptation occurs with different levels of constant background torque, which would differ from the present position-dependent gravity-like torque but could still be exploitable as a motive force. Nevertheless, in the perspective of applications, reproducing arbitrary gravity-like torque at the joints (even approximately) seems to be an appealing paradigm.

## STAR★Methods

### Key resources table

**Table:** 

REAGENT or RESOURCE	SOURCE	IDENTIFIER
**Software and algorithms**		

Python 3.8	Python	https://www.python.org/
C++ 11	Visual Studio Community 2019	https://visualstudio.microsoft.com/
Matlab 2021	MathWorks	https://www.mathworks.com/

**Other**		

ABLE Exoskeleton	Haption	https://www.haption.com/fr/

### Resource availability

#### Lead contact

Further information and requests for resources and reagents should be directed to and will be fulfilled by the lead contact, Dorian Verdel (dorian.verdel@universite-paris-saclay.fr)

#### Materials availability

This study did not generate new unique reagents.

#### Data and code availability

• All data reported in this paper will be shared by the lead contact upon request.
• This paper does not report original code.
• Any additional information required to reanalyze the data reported in this paper is available from the lead contact upon request.

### Experimental model and study participant details

In a series of 3 experiments, a total of 61 healthy participants performed single-joint elbow flexion/extension movements with their forearm in a parasagittal plane, with their upper arm resting in a vertical position as in previous protocols.^29^^,^^46^^,^^87^ The pointing movements thus consisted in point-to-point reaching movements with different gravity-like torques induced at the elbow joint by a robotic exoskeleton.

For all the experiments, written informed consent was given by each participant as required by the Helsinki declaration. Participants’ ethnicity was not collected. The experimental protocols were approved by the local ethical committee for research (Université Paris-Saclay, 2017-34). All the involved participants were right-handed and did not have any known neurological or muscular disorders.

Twenty-two participants were involved in the Experiment 1 (10 females; age: 24±5 years; weight: 70.7±9.7kg; height: 176±7.8cm).

Twenty-nine participants were involved in the Experiment 2 (10 females; age: 23±3 years; weight: 67.1±11.8kg; height: 175±7.6cm).

Ten participants were involved in Experiment 3 (2 females; mean age, 24±3 years; mean weight 69.9±8.7kg; mean height 176±3.5cm).

### Method details

#### Experimental setup

##### Robotic exoskeleton

The experimental conditions described in the present study were achieved with the last actuated axis of the ABLE upper-limb exoskeleton as illustrated in Figure 2A. This exoskeleton has a total of four actuated joints. The first three joints correspond to the three main rotations of the human shoulder (abduction/adduction, internal/external rotation and flexion/extension) and were physically blocked in the present experiment. The last actuated joint corresponds to the flexion/extension of the human elbow. The forearm of the exoskeleton was equipped with a custom-made physical interface, previously developed and validated, minimizing unintended interaction efforts between the exoskeleton and the user.^90^

The different gravity-like compensations were performed by means of a composite control law, based on an open-loop compensation of the exoskeleton dynamics^88^ associated with both an individualized force feedback loop and feedforward terms resulting from a thorough population-based analysis.^46^

##### Kinematic and electromygraphic recording

An optoelectronic tracking system (10 Oqus 500+ cameras, 179Hz; Qualisys, Gothenburg, Sweden) was used to record the position of nineteen 10mm and one 3mm reflective markers placed either on the participant or on the robot. Seven markers were placed on the participant: shoulder (acromion), elbow (epicondyle and epitrochlea), middle of the forearm, wrist (styloid process of the radius), base of the index proximal phalanx and tip of the index finger (the 3mm marker). The other markers placed on the robot were used during the identification process.

The EMG activity was recorded with bipolar surface electrodes (Wave Plus, Wireless EMG, 2000Hz; Cometa, Bareggio, Italy). The QTM interface (Qualisys, Gothenburg, Sweden) allowed recording synchronously kinematic data and EMG activity. Participants were first locally shaved and a hydroalcoholic solution was applied. The electrodes were then placed on the following four muscles: triceps (long and lateral heads), biceps brachii (long head) and brachioradialis. The EMG were placed according to the SENIAM recommendations.^93^

#### Procedures

For each participant, three targets indicated by LEDs were positioned in a parasagittal plane in front of the subject. The central target corresponded to a horizontal position of the forearm and the other two targets were distributed symmetrically with regard to a transverse plane as illustrated in Figure 2A.

##### Experiments 1 and 2

In Experiment 1, each participant was instructed to perform self-paced pointing movements toward semi-spherical targets of 2−cm diameter. Participants were instructed that accuracy was not the primary concern of the task. The movement goal endpoint was the lit LED. The top and bottom LEDs were lit successively, which triggered $45^{\circ }$ flexions and extensions of the human elbow ($\left[-22\text{.}5^{\circ },22\text{.}5^{\circ }\right]$ centered on the horizontal). The LEDs were lit during 1.5s and participants were instructed to complete each movement before they were switched off. This is sensibly longer than the average duration previously observed for movements performed in same conditions with this exoskeleton,^88^ which ensured that participants could move at their preferred velocity. The experiment was divided in eighteen blocks of 15 trials. After a short familiarization with the task outside the exoskeleton, an identification of each participant’s anthropometric parameters was carried out following a preexisting protocol.^46^ This was necessary to accurately implement the desired gravity-like torques. The participants were then asked to perform six blocks inside the exoskeleton in transparent mode, with minimized residual perturbation from the exoskeleton. This condition was referred to as 1g. The participants were then asked to perform six blocks inside the exoskeleton, either in mechanically induced zero-gravity (e.g. $g\approx 0m\text{.}s^{-2}$) or in mechanically induced reversed gravity (e.g. $g\approx +9.81m\text{.}s^{-2}$). These conditions were respectively referred to as 0g and -1g. The order of these two conditions was randomized. Between each block, two-minutes resting breaks were taken, during which the participants were asked not to move their forearm to avoid any readaptation to the Earth’s gravity field.

Experiment 2 essentially followed the same protocol as Experiment 1. The differences were a different number of blocks in each gravity condition (*e.g.* 2 instead of 6) and a higher number of trials per block (*e.g.* 25 instead of 15).

##### Experiment 3

Experiment 3 globally followed the same procedure as Experiment 1. The specific characteristics of this experiment were: the blocks were composed of 15 elbow flexions and 15 elbow extensions as in Experiment 1 and each block was performed with a different mechanically induced gravity-like torque chosen among 11 gravity-like torques with a ±0.2g increment. Half of the participants started the experiment in the 1g condition and were subjected to an increasing gravity-like compensation until they reached the -1g condition. The other half of the participants were subjected to the opposite gradient of gravity-like compensation: they started in the -1g condition and finished in the 1g condition. Between each block the same resting breaks as in Experiment 1 were taken. Experiment 3 was performed with a movement amplitude similar to Experiment 1 (e.g. $45^{\circ }$: $\left[-22\text{.}5^{\circ },22\text{.}5^{\circ }\right]$ centered on the horizontal). Here, we decided to increment or decrement gravity continuously instead of randomly picking a gravity-like torque among 11 possible values to favor the quickness of adaptation given that a restricted number of trials were recorded to limit the duration of the experiment and other effects like fatigue.

### Quantification and statistical analysis

Kinematic and EMG data were processed using custom Python 3.8 scripts.

#### Data processing

##### Kinematics processing

Three-dimensional position data of the marker taped on the tip of the index finger was used to assess the human kinematics. Data from the other reflective markers was used as a control. Position data was filtered (low-pass Butterworth, 5Hz cutoff, fifth-order, zero-phase distortion, ”butter” function from the ”scipy” package) before differentiation as in previous studies.^29^^,^^46^ The computed kinematic parameters were defined as in Figure 2C. The threshold allowing to segment movements was set at 5% of *PV* in agreement with previous studies,^18^^,^^20^^,^^46^ which allowed to define *MD* and movement amplitude.

*PV* and *PA* were respectively defined as the maximum value of the velocity and the maximum positive value of the acceleration reached during each movement. In addition to these absolute parameters, the relative time to peak velocity (rtPV) was computed as the ratio between the time elapsed from the movement onset to *PV* and the duration of the movement.

A movement was considered invalid, and therefore removed, if the acceleration profile crossed 0 more than two times during the movement interval. This led to the exclusion of less than 1% of the movements performed by each participant.

##### EMGs processing

EMG data was first filtered (band-pass Butterworth, [20,450]Hz cutoff, fourth order, zero-phase distortion, ”butter” function from the ”scipy” package), centered and rectified.^94^ Signals were normalized by the maximum peak value from all trials observed during the experiment for each participant and each muscle.^21^^,^^46^^,^^87^ The envelope of the signal was then obtained by filtering the pre-processed signal (low-pass Butterworth, 3Hz cutoff, fifth order, ”butter” function from the ”scipy” package).^94^

A standard procedure was used to separate the tonic and phasic components of the pre-processed EMG signal.^21^^,^^35^^,^^95^ Averaged values of the integrated EMG signal were computed from 1s to 0.5s before the movement began and from 0.5s to 1s after the movement stopped, based on the velocity threshold. The segmentation process based on kinematics recording allowed to ensure that this baseline activity was not affected by the previous and upcoming movement. The tonic component of the EMG was computed as the linear interpolation between these two averaged values. The phasic component of the EMG was computed as the subtraction of the tonic component to the pre-processed signal.

Positive phasic components (referred to as activation), representing muscle bursts exceeding gravity-related EMG level, were assessed by computing the area of the positive phasic activity with a threshold set at 5% of the maximum measured value, which was reached during the acceleration phase. The negative phasic components (referred to as inactivation), were assessed by computing the absolute value of the area of negative phasic activity. A threshold set at −5% of the minimum (negative) measured value was used to detect these negative phasic components. As we dealt with upward movements (i.e. flexions), the activation of the flexors and the inactivation of the extensors were relevant to describe the impact of the various gravity-like torques on muscle patterns. These parameters were computed during the whole movement, without hypotheses on the beginning and end of activation/inactivation.

#### Statistical analysis

Statistical analyses were performed on the averaged values of each participant in each block and/or condition. Normality (*Shapiro-Wilk* test^96^) and sphericity (*Mauchly’s* test^97^) of the distribution of the residuals were verified. Repeated measure analysis of variance (ANOVA) were then performed between the different gravity conditions on the mean values obtained for each participant during Experiment 1 and Experiment 2. These analyses were used to account for differences in kinematics and muscle parameters. Significance of the ANOVA was corrected using a Greenhouse-Geisser method to correct sphericity issues (ϵ<0.75). The significance level of the corrected *p*-value was set at p<0.05.

Pairwise *t*-tests were used to perform post-hoc comparisons. These tests were corrected using Bonferroni method. The significance level of the corrected *p*-value was set at p<0.05. All statistical analyses were performed using custom Python 3.8 scripts and the Pingouin package.^98^

Given the number of participants and conditions in Experiment 3, only quantitative comparisons between the model predictions and the average experimental data were provided there. Mean absolute errors between the model and the experimental data for rtPV, and Pearson correlation coefficients were computed to quantify the agreement between the model and the data when varying gravity incrementally.

#### Simulations

Movement kinematics were predicted using an optimal control model of the human forearm pointing task. Our model implemented the gravity-exploitation theory via the minimization of an effort-based cost considering the external action of the exoskeleton. To this aim, the Smooth-Effort model (*SE*) minimizing a compromise between the absolute work of the elbow net torque and a smoothness regularization term was tested.^10^^,^^29^^,^^99^^,^^100^ The dynamics of the human forearm when taking into account the exoskeleton were simulated as follows:

(Equation 1) $$\left(I+\sigma I_{s}\right)\ddot{\theta }=\tau_{h}+\tau_{r}-B\dot{\theta }-m_{h}l\text{gcos}\left(\theta \right)$$

where *I* was the apparent inertia of the coupled human-exoskeleton system, $I_{s}$ was an inertial shift dependent on movement direction and acceleration phase (see Figure S3 for its rationale and estimated values), σ is either equal to 1 or 0 depending on movement direction and acceleration phase, which allowed to simulate the inertial shifts induced by the exoskeleton (see Figure S3), $\tau_{h}$ was the human net torque at the joint, $\tau_{r}$ was the robot torque, $B=0.05N.m.s\text{.}{\text{rad}}^{-1}$ was the damping of the human elbow^101^ and the product $m_{h}l=0.2934\text{kg}.m$, between the human mass and the weight moment arm, was identified following a preexisting procedure.^46^ The $I=0.35\text{kg.}m^{2}$ term of the apparent inertia was an approximation of the total inertia of the human-exoskeleton system based on human dynamics identification, anthropometric tables and robot dynamics identification.^46^^,^^88^^,^^102^^,^^103^ The robot torque was assumed to be known as it was compensating for the human gravity torque, which allowed rewriting Equation 1 in the different gravity conditions as follows:

(Equation 2) $$\left(I+\sigma I_{s}\right)\ddot{\theta }=\tau_{h}-B\dot{\theta }-m_{h}l\tilde{g}\cos\left(\theta \right)$$

$$\tilde{g}=g-\alpha g,\text{where}\;\alpha \in \left\{0,0.2,\ldots ,1.8,2\right\}$$

Based on these dynamics, the following smoothness term was defined:

(Equation 3) $$C_{s}=\int_{0}^{T}{\left(u_{h}-B\ddot{\theta }+m_{h}l\tilde{g}\dot{\theta }\sin\left(\theta \right)\right)}^{2}dt$$

where $u_{h}={\dot{\tau }}_{h}$ was the human control variable in a commanded torque change framework.^104^ In the *SE* model, the effort term is defined as the absolute work at the human joint, which allows to reproduce the observed inactivation periods,^26^ as follows,

(Equation 4) $$C_{e}=\int_{0}^{T}|\tau_{h}\dot{\theta }|dt$$

In the chosen optimal control framework, motor planning is assumed to originate from the minimization of a cost function, expressed as follows:

(Equation 5) $$J\left(u_{h}\right)=C_{e}+\beta \;C_{s}$$

where β was a weight that allowed adjusting the importance given to each component.

In order to compare the predictions of the model to the data in terms of motor patterns, an analysis of the predicted net torque was conducted. First, this torque was split into a flexors and an extensors contribution, computed as follows,

(Equation 6) $$\text{Flex.}={\left\lfloor \tau_{h}\right\rfloor }_{+}\;\text{and}\;\text{Ext}.=-{\left\lfloor \tau_{h}\right\rfloor }_{-}$$

where ${\left\lfloor \tau_{h}\right\rfloor }_{+}$ is the positive part of the torque and ${\left\lfloor \tau_{h}\right\rfloor }_{-}$ its negative part. Then, the equivalent of flexors activation and extensors inactivation (although they are not quantitatively comparable due to the non-linear muscle dynamics) were respectively computed as the positive and negative areas of the phasic torque normalized by the maximum predicted value. The phasic torque was computed using the same separation method as between tonic and phasic EMG.

All the simulation results reported in the present paper were obtained using the Matlab (MathWorks) version of ”*GPOPS-II*”.^105^^,^^106^^,^^107^ This software is based on an orthogonal collocation method and uses the ”*SNOPT*” solver to solve the nonlinear programming problem.^108^
