# Supplementary material for: Reoptimization of single-joint motor patterns to non-Earth gravity torques induced by a robotic exoskeleton
Source: iScience. 2023 Oct 28;26(11):108350. doi: 10.1016/j.isci.2023.108350 (PMC10665922; doi:10.1016/j.isci.2023.108350)
Supplement: Document S1. Figures S1–S4 [file mmc1.pdf]

iScience, Volume *26*

Supplemental information

Reoptimization of single-joint motor patterns to non-Earth gravity torques induced by a robotic exoskeleton

Dorian Verdel, Simon Bastide, Franck Geffard, Olivier Bruneau, Nicolas Vignais, and Bastien Berret

Figure S1. Behavioral results, related to Figure 4. We investigated the block-wise adaptation that occurred on common kinematic parameters. In particular, potential adaptations were analyzed through movement duration (MD), amplitude, peak velocity (PV), peak acceleration (PA), relative duration of the agonist muscles activation and relative duration of the antagonist muscles inactivation. Regarding EMG parameters, the adaptation was first assessed for the relative activation duration of flexors and the relative inactivation duration of extensors. During Experiment 1, the participants performed a total of 90 upward movements and 90 downward movements (6 blocks $\times$ 15 trials per direction) in each of the 1g, 0g and -1g conditions. The block-wise evolution of these parameters for both movement directions is depicted in Figure S1. A visual inspection might suggest a potential adaptation for MD, PV and PA across blocks. In the case of upward movements, when comparing the first and last blocks using Student's paired $t-$tests, we did not find any significant difference for all gravity conditions regarding all the parameters (MD: $p > 0.36$, A: $p > 0.68$, PV: $p > 0.44$, PA: $p > 0.51$, relative duration of flexors activation: $p > 0.24$ and relative duration of extensors inactivation: $p > 0.16$ for all gravity conditions). In the case of downward movements, when performing the same comparisons as for upward movements, we did not find significant differences for all gravity conditions regarding MD (in all cases: $p > 0.14$), amplitude (in all cases: $p > 0.7$), PV (in all cases: $p > 0.25$), PA (in all cases: $p > 0.27$), and relative duration of the antagonist muscles inactivation (in all cases: $p > 0.73$). However, a significant difference was found for the -1g condition on the relative duration of the agonist muscles activation ($p = 0.02$), which was not reproduced for the other gravity conditions (in both cases: $p > 0.73$). Overall, it can be concluded that participants adapted to the imposed gravity conditions before the end of the first block. Indeed, given the number of performed tests (*i.e.*, 36) and the variability of the analyzed parameters, the only significant difference observed in the -1g condition on the relative duration of the agonist muscles activation cannot be seen as a strong sign of an ongoing long adaptation process. More importantly, clear differences between conditions are observable from the first block on kinematics (in particular PV and PA) and muscle activation and inactivation patterns.

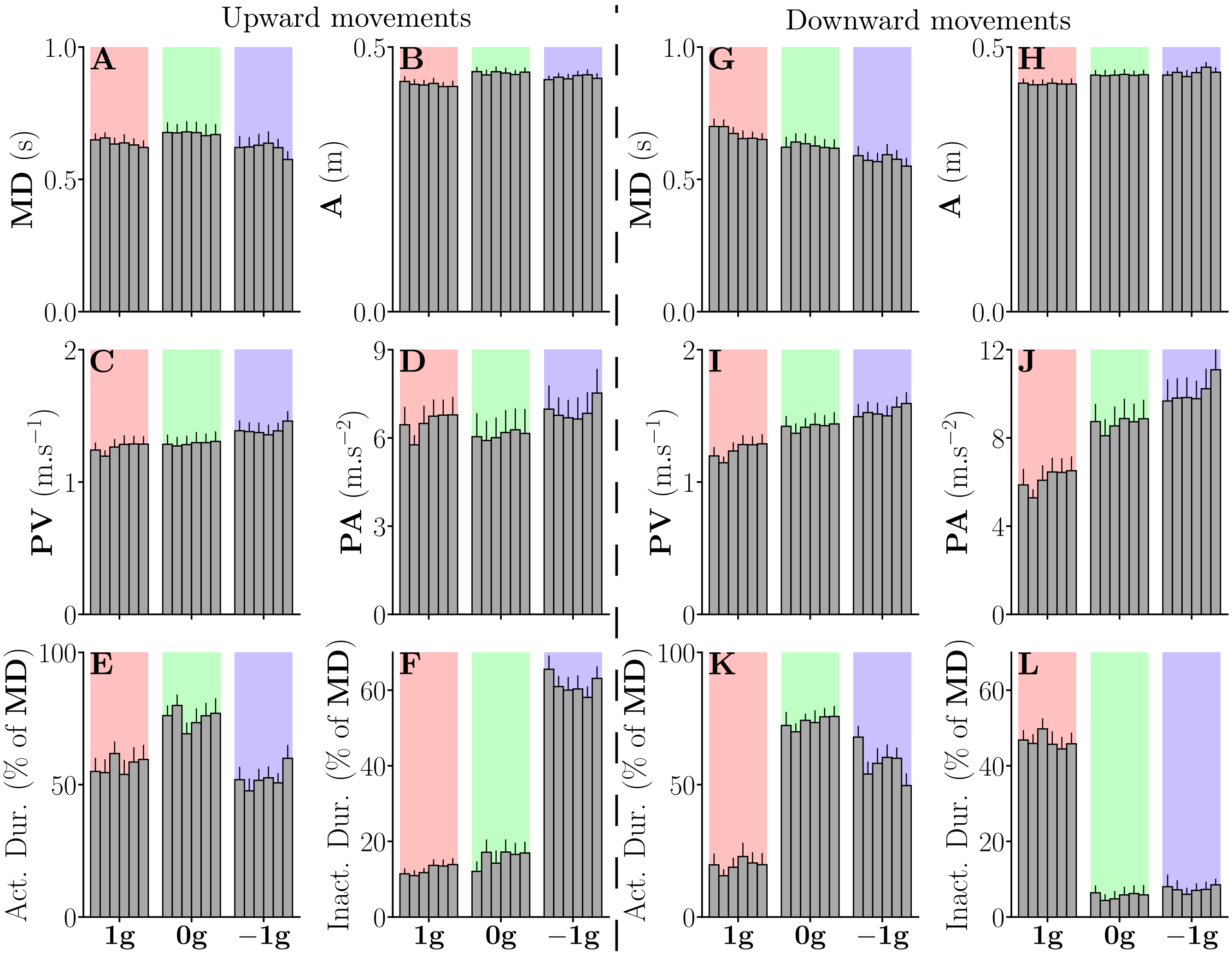

Figure S1. Averaged parameters for each block, error bars represent the standard error across participants and upward movements are described on the left side while downward movements are described on the right side, related to Figure 4. A,G.) Movement duration MD. **B,H.)** Amplitude A. **C,I.)** Peak velocity PV. **D,J.)** Peak Acceleration PA. **E,K.)** Relative duration of the agonist muscles activation. **F,L.)** Relative duration of the antagonist muscles inactivation.

Figure S2. Behavioral results, related to Figure 4. The effect of gravity on movement kinematics being usually assessed from the rtPV and its effect on EMGs from the activation and inactivation of flexors and extensors respectively [S1–S7], we then assessed the adaptation of these parameters (see Fig. S2). Again, on upward movements, paired t-tests did not reveal any difference between the first and the last blocks performed under each gravity condition for the three parameters (rtPV: $p > 0.62$; flexors activation: $p > 0.5$; extensors inactivation: $p > 0.23$ for all gravity conditions). In the case of downward movements, the same comparisons did not reveal any difference between the first and last block on rtPV (in all cases: $p > 0.12$) and flexors inactivation (in all cases: $p > 0.23$). However, consistently with the previous results, the extensors activation exhibited a significant difference between the first and last block in the -1g condition ($p = 0.01$), which was not reproduced in the other gravity conditions (in both cases: $p > 0.84$). All these analyses confirm that no major change in the forearm trajectories

occurred across blocks and that a plateau was quickly attained. In the subsequent analyses, we thus averaged data across blocks and focused on the differences between gravity conditions.

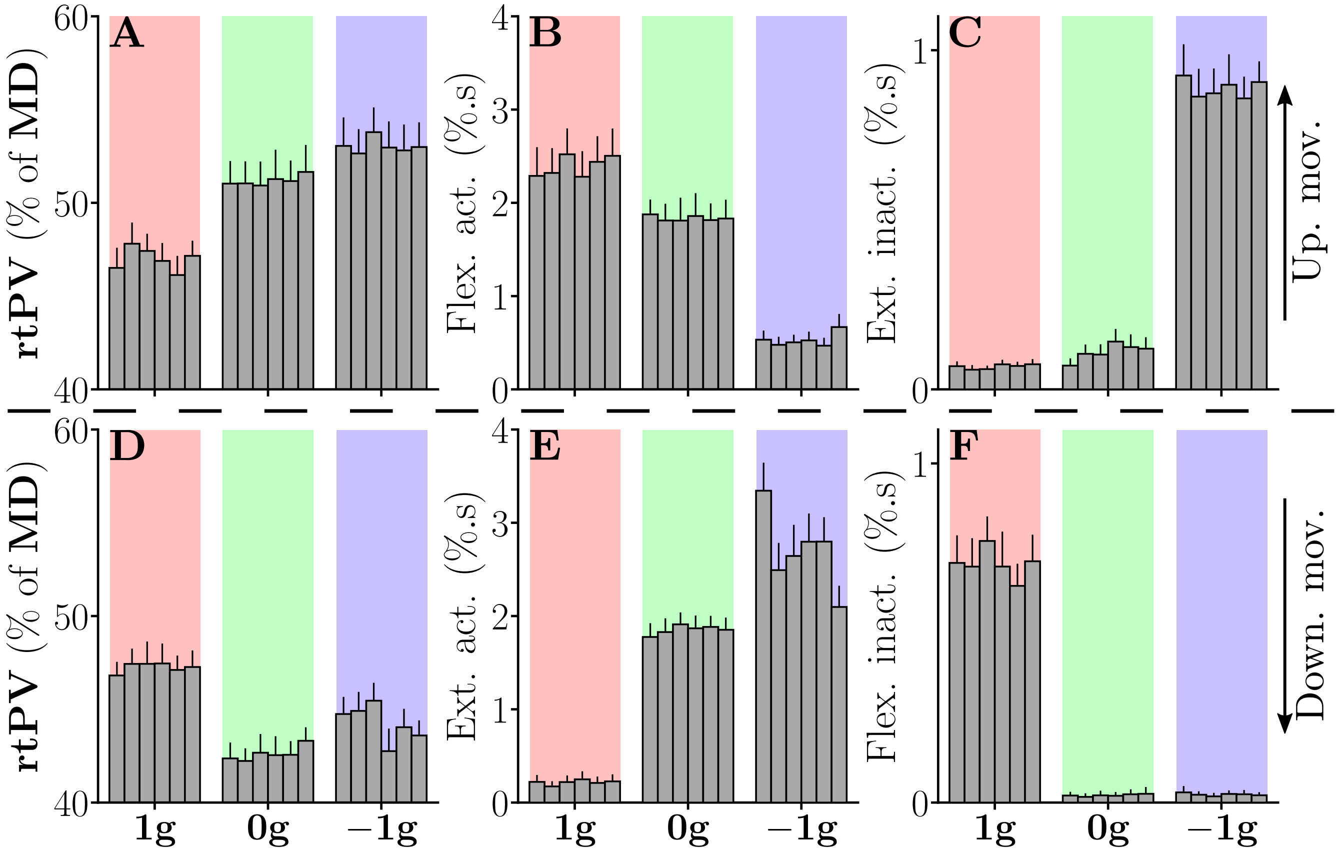

Figure S2. Averaged parameters for each block, error bars represent the standard error across participants and upward movements are described on the first line while downward movements are described on the second line, related to Figure 4. A.) Upward relative time to peak velocity rtPV. **B.**) Flexors activation. **C.**) Extensors inactivation. **D.**) Downward rtPV. **E.**) Extensors activation. **F.**) Flexors inactivation.

Figure S3. Inertial shift, related to STAR Methods. As discussed in the main text of the paper, the inertial effects associated with the exoskeleton could not be fully compensated during the experiments. In particular, this induced a shift from the compensation of the joint effects of gravity. This shift is illustrated in Figure S3 for both upward and downward movements. The difference of shift observed between movement onset (upon which humans plan their movements) and the end of movement could explain the observed shifts in the rtPV when compared to previous studies (for example [S3]). On average, the shift for upward movements was estimated to be around a third of the shift observed for downward movements. For downward movements, a shift equal to $I_s = 0.5I$ was found to be sensitive as it allowed to reproduce the experimental data while remaining mechanically plausible (higher values would attribute too much importance to these efforts). Consequently, the shift for upward movements was set at $I_s = 0.17I$. Such a difference of inertial error between upward and downward movements could be explained by higher tracking delays in the controller for the lasts, which might be due to different motor quadrants between upwards movements (in such movements the motor tracks positive torques with positive velocities) and downward movements (in such movements the motor tracks

positive torques with negative velocities, which is braking). Indeed, such differences in the motor quadrant have been shown to have an impact both on the transmission and on the friction at low velocities (beginning of movement) [S8–S10]. In particular, in the present experiment, frictions are statically compensated before upward movements because the controller tracks a positive (*i.e.*, upwards) torque. On the contrary, before downward movements, frictions are not statically compensated because the controller also tracks a positive torque, which results in a higher resistance and consequently a higher felt inertia at the human elbow level during the beginning of movement (see differences in tracking errors between upwards and downwards in Fig. S3). Then, the controller is able to catch up with the human thanks to the interaction torque feedback control. Such effects of the specific dynamics of our exoskeleton could possibly be mitigated by implementing a step of human intention prediction in future controllers (for example based on a classification of EMG signals [S11]), which would allow to compensate for this higher apparent inertia based on the predicted movement timing and direction.

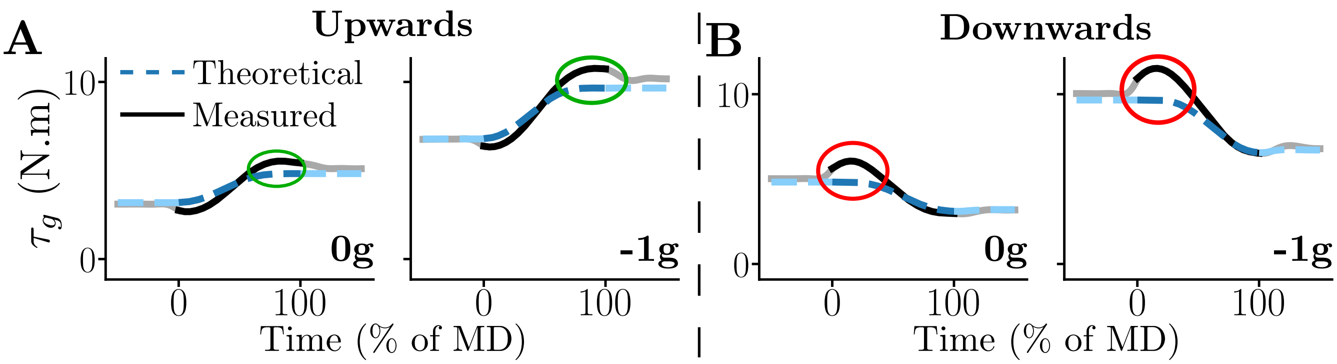

Figure S3. Illustration of the inertial shift due to the robot control and dynamics, related to STAR Methods. A.) Upward movements. **B.**) Downward movements.

Figure S4. Dependence of rtPV on MD, related to STAR Methods. As stated in the present and previous studies [S3, S6, S12], the rtPV is known to be impacted by gravity. However, this parameter is also sensitive to MD. Consequently, it is necessary to assess whether variations in MD could explain the experimental data when participants were submitted to a gradient of gravity-like torques. Therefore, we computed the rtPV of simulated movements for a large range of MD (including all the experimental MD), which results are reported in Figure S4. These simulation results clearly show that the predicted rtPV when modulating MD cannot reproduce the experimental data of Experiment 3 and the predictions associated with a gradual change of rtPV provided in Figure 1. Indeed, the population data are either above or under the rtPV predicted when modulating MD. In contrast, the experimental variations in rtPV observed during Experiment 3 can be accurately predicted by varying the local gravity-like torque.

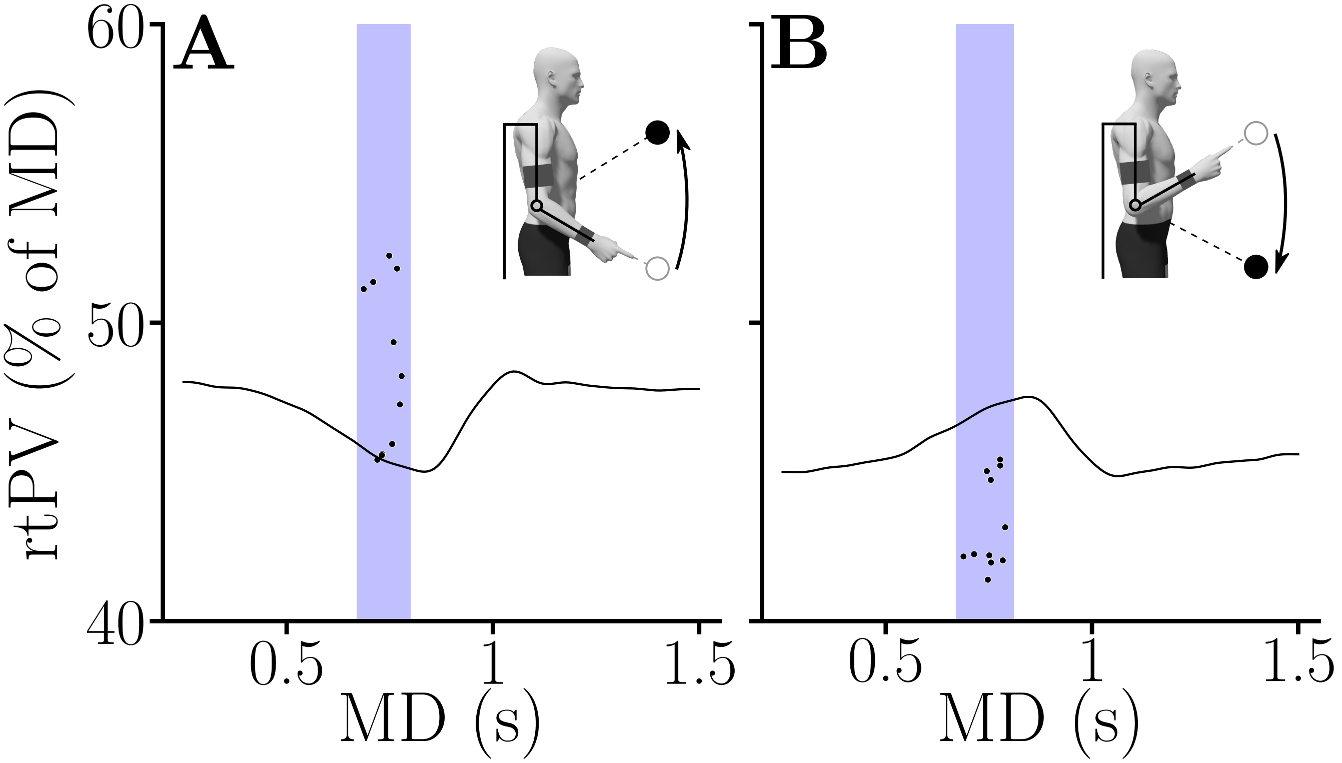

Figure S4. Illustration of the effect of MD on the rtPV for simulations performed with the unifying value of $\beta = 18 \times 10^{-4}$ minimizing the AAE for both upward and downward movements, the blue shaded area corresponds to the interval of experimental MD and the black dots represent the population average rtPV with respect to MD measured during Experiment 3, related to STAR Methods. A.) Simulated upward movements. **B.**) Simulated downward movements.

References

[S1] Crevecoeur, F., Thonnard, J.-L., and Lefèvre, P. (2009). Optimal integration of gravity in trajectory planning of vertical pointing movements. Journal of Neurophysiology 102, 786–796.

[S2] Gaveau, J., and Papaxanthis, C. (2011). The temporal structure of vertical arm movements. PLoS ONE 6, e22045.

[S3] Gaveau, J., Berret, B., Demougeot, L., Fadiga, L., Pozzo, T., and Papaxanthis, C. (2014). Energy-related optimal control accounts for gravitational load: comparing shoulder, elbow, and wrist rotations. Journal of Neurophysiology 111, 4–16.

[S4] Gaveau, J., Berret, B., Angelaki, D. E., and Papaxanthis, C. (2016). Direction-dependent arm kinematics reveal optimal integration of gravity cues. eLife 5, 1–17.

[S5] Gentili, R., Cahouet, V., and Papaxanthis, C. (2007). Motor planning of arm movements is direction-dependent in the gravity field. Neuroscience 1, 20–32.

[S6] Gaveau, J., Grospretre, S., Berret, B., Angelaki, D. E., and Papaxanthis, C. (2021). A cross-species neural integration of gravity for motor optimization. Science Advances 7, eabf7800.

[S7] Poirier, G., Papaxanthis, C., Mourey, F., Lebigre, M., and Gaveau, J. (2022). Muscle effort is best minimized by the right-dominant arm in the gravity field. Journal of Neurophysiology 127, 1117–1126.

[S8] Garrec, P., Friconneau, J.-P., Méasson, Y., and Perrot, Y. (2008). ABLE, an Innovative Transparent Exoskeleton for the Upper-Limb. IEEE/RSJ International Conference on Intelligent Robots and Systems (IROS) (1483–1488).

[S9] Garrec, P. (2010). Screw and Cable Acutators (SCS) and Their Applications to Force Feedback Teleoperation, Exoskeleton and Anthropomorphic Robotics. Robotics 2010 Current and Future Challenges (167–191).

[S10] Hamon, P., Gautier, M., and Garrec, P. (2010). Dynamic identification of robots with a dry friction model depending on load and velocity. IEEE/RSJ International Conference on Intelligent Robots and Systems (IROS) (6187–6193).

[S11] Trigili, E., Grazi, L., Crea, S., Accogli, A., Carpaneto, J., Micera, S., Vitiello, N., and Panarese, A. (2019). Detection of movement onset using EMG signals for upper-limb exoskeletons in reaching tasks. Journal of NeuroEngineering and Rehabilitation 16.

[S12] Papaxanthis, C., Pozzo, T., and Stapley, P. (1998). Effects of movement direction upon kinematic characteristics of vertical arm pointing movements in man. Neuroscience Letters 253, 103–106.
